# A systematic assessment of the concept and practice of public-private mix for tuberculosis care and control

**DOI:** 10.1186/1475-9276-10-49

**Published:** 2011-11-10

**Authors:** Rasmus Malmborg, Gillian Mann, S Bertel Squire

**Affiliations:** 1 International Department, The Norwegian Hearth and Lung Patient Organisation, LHL,(Storgata 33), Oslo, (0184), Norway; 2 Collaboration for Research on Equity and Systems in TB and HIV-AIDS (CRESTHA), Liverpool School of Tropical Medicine, (Pembroke Place), Liverpool (L3 5QA), UK

**Keywords:** Tuberculosis, Public-Private Mix, case detection, treatment outcome, equity, access, cost

## Abstract

**Introduction:**

The STOP TB Partnership aims to improve global tuberculosis (TB) control through expanding access to the directly observed treatment short course (DOTS) strategy. One approach to this is 'Engaging all Care Providers', which evolved from 'Public-Private Mix (PPM) DOTS'. The overall aim of this study was to systematically assess whether and to what degree the STOP TB Partnership's four global objectives of engaging all care providers are met through existing PPM interventions. These four objectives are; 1) Increase TB case detection; 2) Improve TB treatment outcomes; 3) Enhance access and equity; 4) Reduce financial burden on patients. The specific objectives of this assessment were to 1) Understand what PPM means to the STOP TB Partnership's PPM Subgroup and to National Tuberculosis Programme managers; 2) Scope the nature of existing country-level PPM interventions and 3) Review PPM practice against the global PPM objectives.

**Methods:**

We undertook a systematic, multi-facetted assessment. The methods included **i**nterviews with National Tuberculosis Programme managers from high burden countries, clarification of key issues with the STOP TB Partnership PPM secretariat and a review of publicly accessible reports and published articles on PPM projects. Both the literature review and interviews with the National Tuberculosis Programme managers yielded data on project characteristics; PPM models at country level; National Tuberculosis Programme partners; and mechanisms for engagement. Matrices were developed from the literature review and the interviews to show the relationship between services and service providers for different PPM projects. Data from the literature were assessed against each of the four global PPM objectives.

**Results:**

Twelve National Tuberculosis Programme managers from high burden countries were interviewed about the scope of PPM partnerships. Understanding of PPM and types of engaged providers varied considerably; 'private-for-profit qualified clinical providers' were the dominant category. The literature review yielded information on 22 projects in which 'private-for-profit qualified clinical providers' were again the dominant category. The contributions made by 'private-for-profit qualified clinical providers' and 'Non Governmental Organisation qualified clinical providers', were assessed against the four global PPM objectives. Reporting on tuberculosis case detection and treatment outcomes was generally good and demonstrated important PPM contributions in these areas. Reporting on equity, access and reduced patient costs was often lacking or inconclusive.

**Conclusions:**

PPM has improved case detection and treatment outcomes among patients seeking care with private providers. Evidence on reducing patient costs is inconclusive, and there is scope for increasing equity in access to care by systematically engaging those providers who are the primary agents for poor people seeking health care. Guidelines outlining which types of providers best contribute to achieving the four global objectives, along with the resources required by National Tuberculosis Programs for such engagement is needed.

## Introduction

Tuberculosis (TB) causes 1.8 million deaths annually. The majority of cases are found in low or low-middle income countries[1] and studies in both high-income[2] and low-income[3] countries demonstrate significantly higher rates of TB in their poorer populations.

Directly observed treatment short course (DOTS) was introduced as the Global Strategy to address TB in 1994[4]. DOTS comprises five key components: 1) Political commitment; 2) Case detection through quality-assured bacteriology; 3) Standardized treatment with supervision and patient support; 4) An effective drug supply and management system; 5) Monitoring and evaluation system and impact measurement [5]. It is still seen as the corner stone of TB control today. Its success is largely measured through case detection and treatment success rates. Case detection requires that TB is primarily diagnosed in a patient through bacteriology and is reported within the national surveillance system and hence to WHO[6]. Treatment success is achieved when a patient who was sputum smear positive completes treatment and is cured (they become smear negative) or when a patient who was smear negative completes treatment [7]. Originally DOTS was primarily implemented through National Tuberculosis Programmes (NTPs). It was recognised, however, that health systems are pluralistic and that private practitioners (often general practitioners) functioning in isolation from NTPs were an important source of care for many patients but that their services did not meet international standards[8]. The potential for engaging private providers for TB control was described in Uplekar et al, 2001[9]. By 2003 a strategy, known as Public-Private Mix DOTS (PPM DOTS), for engaging private providers in order to improve TB control had been established[10] and by 2006 was supported by the International Standards for TB Care[11]. During the subsequent few years the PPM DOTS concept expanded to encompass engagement with a range of providers, including some semi-qualified providers[12], traditional providers[13] and public and private hospitals[14,15]. PPM DOTS is now known as 'PPM for TB Care and Control' and is a core component of the WHO STOP TB Strategy, entitled 'Engage All Care Providers'[16] and supported by a toolkit to assist implementation[17].

**Figure 1:**
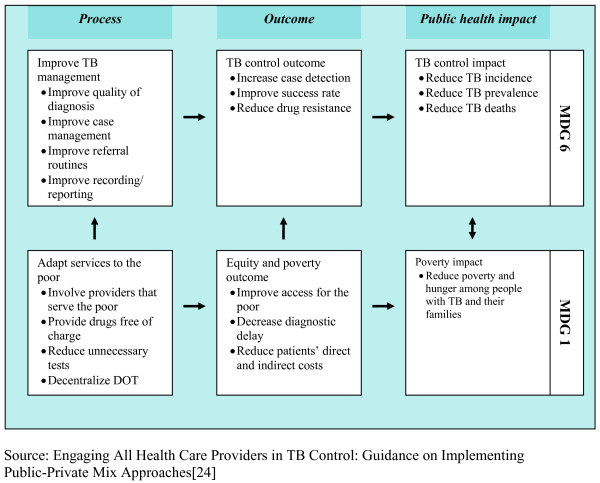
**Logical framework for linkages between PPM objectives, Tuberculosis (TB) control and Millennium Development Goals (MDGs)**. Source: Engaging All Health Care Providers in TB Control: Guidance on Implementing Public-Private Mix Approaches[24].

PPM for TB Care and Control (PPM) is by definition a complex, context-specific, health system intervention[18] and it is seen as a catalyst[19,20] for meeting the WHO and Stop TB Partnership targets for global TB control[16,21-23]. Guidelines in 2006 for implementing PPM[24] stressed the need for NTPs to set their own objectives but to link these to the Millennium Development Goals (MDGs). They also illustrated the mechanisms through which PPM can facilitate attainment of the MDGs (shown in Figure 1). The most recent PPM toolkit has now developed this thinking further and articulates specific PPM objectives[17]:

1) Increase TB case detection

2) Improve TB treatment outcome

3) Enhance access and equity

4) Reduce financial burden on patients

There have been strong calls for evaluations of health system interventions like PPM in order to promote further policy and practice development[25,26]. Several evaluations and cross project comparisons of PPM projects have been published [27-31], primarily focusing on case notifications and treatment outcomes. Some studies assessing the cost effectiveness of individual PPM projects have also been published[32-35]. While these have been helpful in assessing whether PPM can increase case notifications and improve treatment success, they have not attempted to take a comprehensive, systematic approach to synthesising all the evidence, they have not addressed the other PPM objectives, nor assessed which types of provider are effective at addressing which of the objectives. Our aim was to understand the range of PPM interventions (including but not limited to those reported through peer reviewed publications) and systematically to assess whether, and to what degree, these global objectives are met through existing PPM interventions.

The phrase 'systematic assessment' was chosen because we have tried to mirror the concept used in systematic reviews [36-38], the specific approach used in this review is adapted from a systematic review by Bosh-Capblanch and Garner [39].

## Methods

The review comprised three objectives:

• Understanding what PPM for TB care and control means to the STOP TB Partnership's PPM Subgroup and to NTP managers

• Mapping the scope of country-level PPM interventions

• Reviewing practice against global PPM objectives

### Understanding what PPM for TB care and control means to the STOP TB Partnership's PPM Subgroup and to NTP managers

The websites and publications of WHO and the STOP TB Partnership were searched for definitions of PPM in May 2009. Further clarifications concerning PPM's specific objectives were sought by email from the STOP TB Partnership's PPM secretariat in November 2009. The websites and publications were checked again in November 2010.

**Figure 2:**
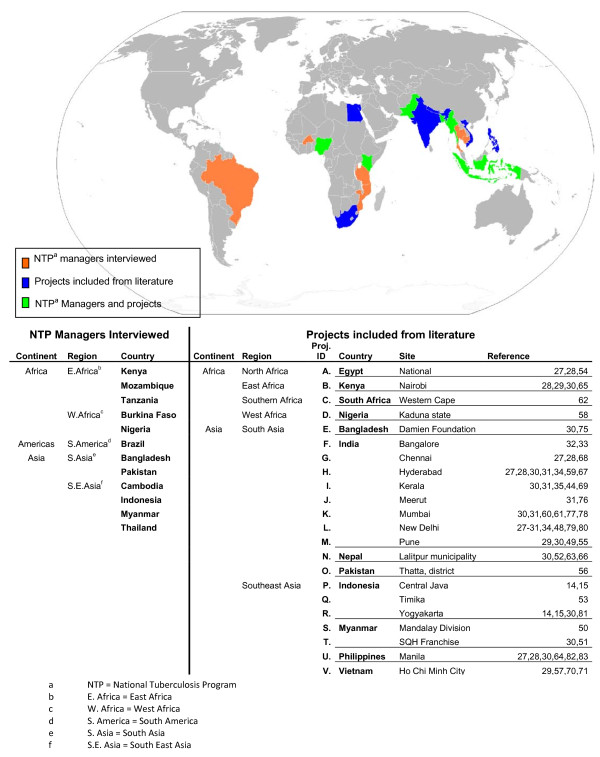
**Countries represented in the study through NTP manager interviews and published literature **[75-83].

We conducted interviews with 12 of the 22 NTP managers from high burden countries during the DOTS Expansion Working Group (http://www.stoptb.org/wg/dots_expansion) meeting in October 2009, to learn about the scope of PPM at country level, the kind of providers engaged and what activities are undertaken by each partner. Convenience sampling was used for the selection of NTP managers, while ensuring representation from each continent (see Figure 2). Summaries of key discussion points and 'PPM diagrams' showing the relationships between different PPM partners were drawn up (based on a previously published template[40]) and sent back to the respective NTP manager for comments. Five responded with comments and/or corrections. After amendment, the diagrams were sent back for final approval, which was received from all.

### Mapping country-level PPM interventions

This comprised the following steps, each of which is discussed in detail below:

a) Developing selection criteria for the literature review

b) Sourcing, reviewing and collating documents and the results of the NTP managers' interviews

c) Developing two matrices outlining actors and services - one from the literature reviews, one from the interviews

#### a) Selection, inclusion and exclusion criteria for literature review

The global objectives of PPM were not articulated before the PPM subgroup of the DOTS Expansion Working Group (henceforth the PPM Subgroup) was established in 2000[41]. The literature search was therefore limited to material published after January 2000 and up to 7^th ^November 2010, using the following search phrases; words where not searched individually:

'Public Private Mix'; 'PPM TB'; 'PPM Tuberculosis'; 'Public Private Mix TB'; 'Public Private Mix Tuberculosis'; 'Private health care provision TB'; 'Private health care provision Tuberculosis'; 'access TB'; 'access Tuberculosis'.

English language abstracts from all articles were initially included as were relevant publicly accessible reports, not found in the peer-reviewed press.

The following exclusion criteria were applied to the abstracts:

• Papers which did not focus on PPM models of TB care provision

• Documents which were not available in their full text form from the UK universities library network

**Figure 3:**
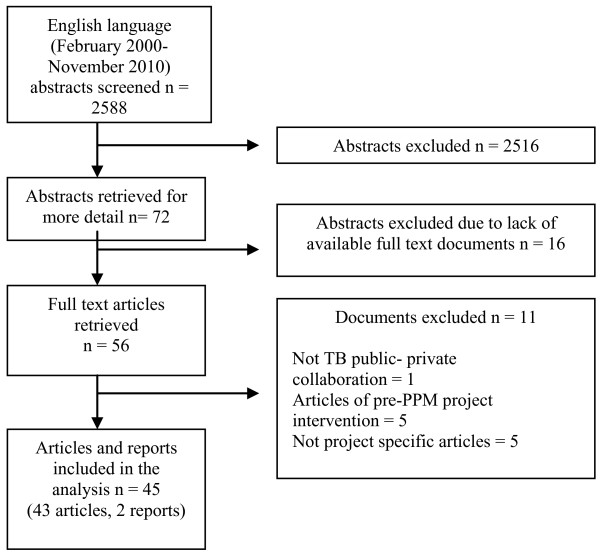
**Process used for literature screening and selection**.

Reference lists of full text articles were screened for relevant references, with the same selection and exclusion criteria applied. Five of the resulting full text articles reviewed were excluded because they did not focus on implemented PPM projects. Figure 3 shows that the search criteria led to an initial discovery of 2,588 abstracts, while the exclusion criteria narrowed these down to 45 relevant articles and reports.

#### b) Sourcing, reviewing and collating documents

The 45 documents were reviewed and grouped by project. If more than one of the documents discussed different aspects of the same PPM project they were grouped as one. Twenty-two projects were identified and are listed in Figure 2. They are named by the country and/or site in which they were performed. They will hereafter be known as the 'projects'.

#### c) Development of matrices

**Figure 4:**
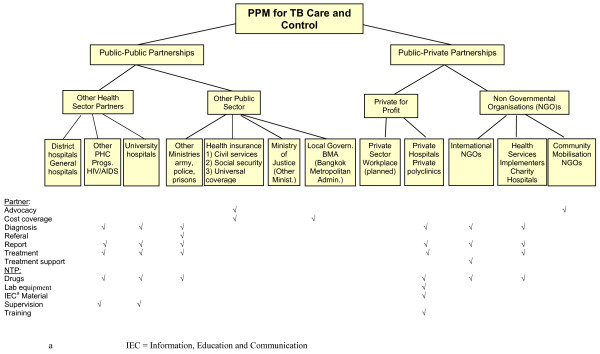
**Nature and scope of PPM for TB Care and Control: example from Thailand**.

The PPM diagrams from the interviews served as a template for mapping providers and services identified through the literature. The Thai PPM model, shown in Figure 4, is an example.

Terminology regarding partners, the services they delivered and the inputs they received differed across the publications and between NTP managers (known collectively henceforth as our sources), we therefore grouped these into common themes, which we termed 'aggregated' partners, services or inputs. For partners of TB programmes these were: other public sector heath partners (PSHP), other public sector partners (OPS), private-for-profit partners (PFP) and non-governmental organisations (NGOs). For clarity, a 'qualified doctor' was defined as someone qualified in bio-medicine. Practitioners with other types of qualifications are, for the purpose of this review, categorised as unqualified practitioners.

Services delivered were aggregated into 15 categories ranging from advocacy to patient follow up. Finally, NTP inputs were collapsed into 12 categories ranging from provision of advocacy, communication and social mobilisation (ACSM) to actual building of a TB clinic. For details on how specific partners, services, or inputs were grouped, see additional files 1, 2,3.

A matrix was constructed representing partners in columns with services and inputs in rows.

#### d) Using the matrix to assess PPM projects

This matrix was completed once from the NTP manager interviews (Table 1) and a second time from the literature (Table 2). A mark was put in the matrix each time a source identified that a partner was engaged in delivering a specific service or the NTP provided a specific input. For each type of partner the total number of marks across all services and inputs was calculated to yield a partner 'score'.

**Table 1:** Matrix illustrating extent of engagement of different partner groups from analysis of NTP manager interviews

	**PSHP**^**a**^				**OPS**^**b**^					**PFP**^**c**^				**NGO**^**d**^			
	**Qual clini serv**^**e**^	**Spec. refer serv**^**f**^	**Other ver. Prog**^**g**^	**Semi-public serv**^**h**^	**Min. provid comm living facil**^**i**^	**Min. of social secur**^**j**^	**Health insurance**	**Other ministries**	**Para-statals**	**Qual clini serv**^**e**^	**Qual ancill health serv**^**k**^	**Informal providers**	**Work-place programmes**	**Qual clini serv**^**e**^	**Internationnal NGOs**	**Informal un-qualified providers**	**TOTAL SCORE**
																	
SERVICES																	

Advocacy	3	0	3	0	2	1	1	2	3	**1**	0	1	3	2	1	4	27

Active suspect identification	0	0	0	0	0	0	0	0	0	**0**	0	0	0	0	0	1	1

**Diagnosis**	**9**	**1**	**6**	**0**	**11**	**1**	**0**	**2**	**4**	**12**	**4**	**0**	**6**	**9**	**2**	**1**	**68**

Referral	1	0	1	0	3	1	0	2	2	**4**	2	1	1	1	0	3	22

Treatment	9	2	6	0	11	1	0	2	4	**12**	2	0	5	9	1	2	66

Health Education	0	0	0	0	0	0	0	0	0	**0**	0	0	0	1	0	0	1

Reporting	9	2	6	0	10	1	0	1	3	**12**	4		5	8	2	2	65

DOT^l^	1	0	0	0	0	0	0	0	0	**1**	0	0	0	1	0	0	3

Treatment support	0	0	0	0	0	1	0	1	0	**0**	0	1	2	0	1	5	11

Defaulter tracing	0	0	0	0	0	0	0	0	0	**1**	0	0	0	1	0	0	2

Political lobbying	0	0	2	0	0	0	0	3	0	**0**	0	0	2	0	0	0	7

Follow up of work places	0	0	0	0	0	1	0	0	0	**0**	0	0	0	0	0	0	1

**TOTAL SCORE**	32	5	24	0	37	7	1	13	16	**43**	12	3	24	32	7	18	

INPUTS																	

ACSM/IEC^m^	1	0	0	0	0	0	0	0	1	**1**	0	0	0	1	0	0	4

BCG^n^	1	0	1	0	0	0	0	0	1	**0**	0	0	0	0	0	0	3

Diagnosis	0	0	0	0	0	0	0	0	0	**0**	0	0	0	0	0	0	0

**Drugs**	**4**	**0**	**3**	**0**	**9**	**1**	**0**	**2**	**4**	**9**	**1**	**0**	**3**	**7**	**2**	**2**	**47**

Defaulter tracing	0	0	0	0	0	0	0	0	0	**0**	0	0	0	0	0	0	0

Pay for service	0	0	0	0	1	0	0	0	0	**1**	1	0	0	1	1	0	5

Diagnostic supplies	2	0	1	0	5	0	0	0	2	**6**	0	0	2	7	0	0	25

Monitoring supplies	1	0	0	0	1	0	0	0	0	**2**	0	0	0	1	0	1	6

Supervision	4	1	2	0	4	0	0	0	1	**3**	1	0	2	5	0	2	25

Capacity building	3	0	1	0	6	1	0	0	2	**5**	1	1	3	5	0	2	30

Transport	0	0	0	0	1	0	0	0	0	**0**	0	0	0	0	0	0	1

TB-clinic (building)	0	0	0	0	1	0	0	0	0	**0**	0	0	0	0	0	0	1

**TOTAL SCORE**	16	1	8	0	28	2	0	2	11	**27**	4	1	10	27	3	7	

NB: Highest ranking provider group, service type and input type are highlighted in bold.

a PSHP = Public Sector Health Partner

b OPS = Other Public Sector

c PFP = Private for Profit

d NGO = Non Governmental Organisation

e Qual. clin. serv. = Qualified clinical services

f Spec. refer serv. = Specialist referral services

g Other ver. prog = Other vertical programmes

h Semi public serv. = Semi-public services

i Min. provid. comm living facil = Ministries providing communal living facilities

j Min. of social secure = Ministry of social security

k Qual ancill health serv = Qualified ancillary health services

l DOT = Directly Observed Treatment

m ACSM/IEC = Advocacy, Communication and Social Mobilisation/Information, Education and Communication

n BCG = Bacille Calmette Guérin vaccine

**Table 2:** Matrix illustrating extent of engagement of different provider groups from analysis of literature.

	**PSHP**^**a**^				**OPS**^**b**^					**PFP**^**c**^				**NGO**^**d**^			
	**Qual clini serv**^**e**^	**Spec. refer serv**^**f**^	**Other ver. Prog**^**g**^	**Semi-public serv**^**h**^	**Min. provid comm living facil**^**i**^	**Min. of social secur**^**j**^	**Health insurance**	**Other ministries**	**Para-statals**	**Qual clini serv**^**e**^	**Qual ancill health serv**^**k**^	**Informal providers**	**Work-place programmes**	**Qual clini serv**^**e**^	**Internationnal NGOs**	**Informal un-qualified providers**	**TOTAL SCORE**
																	
SERVICES																	

Advocacy	0	0	0	0	0	1	0	1	0	**0**	0	0	0	3	1	2	8

Active suspect identification	1	0	0	0	0	0	0	0	0	**2**	0	1	0	2	0	2	8

Diagnosis	5	5	1	1	2	0	1	0	0	**13**	5	1	2	8	0	0	44

Referral	4	3	1	0	1	0	0	0	0	**13**	2	3	2	4	0	2	35

**Treatment**	**5**	**5**	**1**	**1**	**2**	**0**	**1**	**0**	**0**	**16**	**1**	**4**	**2**	**8**	**0**	**0**	**46**

Health Education	0	1	0	0	1	1	1	0	0	**0**	0	0	0	2	0	2	8

Reporting	5	4	1	1	2	0	1	0	0	**15**	1	3	2	8	0	1	44

DOT^l^	0	0	0	1	0	0	0	0	0	**3**	0	1	0	3	0	5	13

Treatment support	0	0	0	0	0	0	0	0	0	**1**	0	1	0	0	0	5	7

Defaulter tracing	1	0	0	0	0	0	1	0	0	**1**	0	1	0	7	0	4	15

Political lobbying	0	0	0	0	0	1	0	0	0	**0**	0	0	0	0	0	0	1

Follow up of work places	0	0	0	0	0	0	0	0	0	**0**	0	0	0	0	0	0	0

**TOTAL SCORE**	21	18	4	4	8	3	5	1	0	**64**	9	15	8	45	1	23	

INPUTS																	

ACSM/IEC^m^	0	0	0	0	0	0	0	0	0	**0**	0	0	0	1	0	1	2

BCG^n^	0	0	0	0	0	0	0	0	0	**0**	0	0	0	0	0	0	0

Diagnosis	0	0	0	0	0	0	0	0	0	**2**	0	0	0	2	0	2	6

**Drugs**	**2**	**3**	**0**	**1**	**1**	**0**	**0**	**0**	**0**	**14**	**1**	**3**	**1**	**5**	**0**	**2**	**33**

Defaulter tracing	0	1	0	0	0	0	0	0	0	**3**	1	0	0	1	0	0	6

Pay for service	0	0	0	0	0	0	0	0	0	**0**	1	0	1	0	0	0	2

Diagnostic supplies	1	1	0	0	0	0	0	0	0	**5**	0	2	0	3	0	0	12

Monitoring supplies	1	2	0	0	0	0	0	0	0	**7**	1	2	1	3	0	1	18

Supervision	2	3	0	1	0	0	1	0	0	**9**	4	1	0	2	0	0	23

Capacity building	2	4	0	1	1	1	1	0	0	**13**	4	1	0	3	0	2	33

Transport	0	0	0	1	0	0	0	0	0	**1**	0	0	0	0	0	0	2

TB-clinic (building)	0	0	0	0	0	0	0	0	0	**0**	0	0	0	0	0	0	0

**TOTAL SCORE**	8	14	0	4	2	1	2	0	0	**54**	12	9	3	20	0	8	

NB: Highest ranking provider group, service type and input type are highlighted in bold.

a PSHP = Public Sector Health Partner

b OPS = Other Public Sector

c PFP = Private for Profit

d NGO = Non Governmental Organisation

e Qual. clin. serv. = Qualified clinical services

f Spec. refer serv. = Specialist referral services

g Other ver. prog = Other vertical programmes

h Semi public serv. = Semi-public services

i Min. provid. comm living facil = Ministries providing communal living facilities

j Min. of social secure = Ministry of social security

k Qual ancill health serv = Qualified ancillary health services

l DOT = Directly Observed Treatment

m ACSM/IEC = Advocacy, Communication and Social Mobilisation/Information, Education and Communication

n BCG = Bacille Calmette Guérin vaccine

The 'score' was used as a proxy measure of engagement. Thus the highest 'score' among the types of providers indicated which provider type is most commonly engaged, the highest 'score' of services indicated which service type was the most commonly given by the providers, and the highest 'score' of inputs indicated which input type was the most commonly received by the providers. The matrices thus provided an overview of the frequency with which an aggregated partner group provided an aggregated service type, and received different aggregated inputs. The groups which appeared with the greatest frequency were then selected for the final stage of analysis: reviewing against global objectives.

### Reviewing practice against global PPM objectives

The PPM global objectives are stated in terms of improved outcomes. In order to assess whether PPM interventions have met these objectives it was important to be clear whether a) the project publications presented data relating to the outcomes and b) the study designs enabled improvements in outcomes to be reasonably attributed to the PPM interventions. We recognised that the publications of the interventions did not set out to answer these questions explicitly and that in some cases measurement against these objectives was not considered a necessary part of the intervention design or assessment. We therefore had to establish some criteria to assess whether each publication presented data of sufficient quality (for our purposes) to undertake such a review.

We were keen to use a standardized tool for assessing the quality of studies and so reviewed a number that are employed in health and health systems research. The nature of PPM as a complex intervention, however, made this challenging. For example the Quality Assessment of Diagnostic Accuracy Studies (QUADAS) [42] tool was designed for diagnostics and clinical research and simple interventions. A range of tools developed by the Critical Appraisal Skills Programme (CASP) [43] were also considered but required the evaluator to know *a priori *the detailed study design. In most of the 22 projects reviewed, the interventions were usually clearly described, but the study design used to measure the effect was not explicitly stated, making the use of CASP problematic. For example, in Kannur District, India, private laboratories were engaged. The NTP gave free training and quality assurance supervision. Numbers of cases detected by the private laboratories and their treatment outcomes were compared to numbers of cases detected in the public sector. Total numbers of TB cases detected in the 18 months prior to the intervention were compared with numbers detected in the 18 months after the intervention was implemented. This is, therefore, an observational study with a before-and-after comparison, but this is not stated *a priori *[44].

Experts in systematic reviews for complex interventions were also consulted to identify the most appropriate review method.

The authors adapted common concepts from the tools noted above to develop a data quality checklist comprising the following dichotomous questions:

• Are any data presented?

• Are data compared to a study control arm?

• Are data compared to non-engaged private sector providers in the locality?

• Are data compared to public sector providers in the locality?

• Are data compared to country/regional data?

• Are there contemporaneous comparisons for any of the above?

The authors developed review questions for each of the global PPM objectives and used each question to assess the literature against the data quality check list above (see Table 3)

**Table 3:** Data availability for review against Global PPM objectives

	Any data presented	Data compared to:				Data compared to other involved providers in locality	Projects for which any quantitative data presented*
Numbers of programmes for which:		Study control arm	Respectively Non-engaged private or NGO^a ^providers in locality	Public sector in locality	Country/Regional data		
**PFPs^b^**							
Increase case detection	16	4	2	14	11	10	A,B,F†,H,I,J,K,L,M,N,O,R†,S,T,U,V
Improve Treatment Outcome	14	4	2	12	8	10	F†,B,H†,I,J,K,L,N†,P,R,S,T,U†,V
Enhance Access and Equity	8	3	4	5	1	6	F†,H,K,L,N,S,T,V
Reduce financial burden on patients	7	4	7	5	1	7	F†,H,K,L,S,T,V
**NGOs^a^**							
Increase case detection	8	0	1	4	4	4	A,C,F,H,J,N,Q†,U
Improve Treatment Outcome	9	0	1	6	6	4	C,D,F,H,J,N,Q†,U
Enhance Access and Equity	4	0	1	2	0	2	C,F†,H,N
Reduce financial burden on patients	2	0	1	2	0	2	F†,H

* Each letter identifies an individual project; it is the project i.d. for list of projects, please see figure 3

† Data are not disaggregated between different types of providers

a NGO = Non Governmental Organisation

b PFP = Private For Profit

1.) Increase case detection

a.) Does the involvement of a given partner group increase case detection?

2.) Improve treatment outcome

a.) Does the involvement of a given partner group improve treatment outcome?

3.) Enhance access and equity

a.) Is the partner group geographically located in areas where the population is predominantly poor?

b.) Does it provide services to demonstrable poor (e.g. through assets measures), vulnerable or other marginalised population groups?

c.) Has the engagement of the partner group altered the proportion of women and men who access services?

4.) Reduced financial burden on patients

a.) Does the involvement of the partner group reduce the financial burden on patients?

b.) Do poor people (defined by local poverty measures) benefit from lower cost of treatment when the partner group is engaged?

The most challenging of these was assessing equity in interventions where this was not specifically an objective of the individual projects. To do this as systematically as possible, we followed the seven steps recommended for assessment of equity in systematic reviews[45]. The steps, along with a descriptor of how we achieved each step, are outlined below:

(1) Developing a logic model: the construction of the matrices

(2) Defining disadvantage and for whom interventions are intended: the authors chose a definition of 'the poor' comprising three key indicators[40,46]:

a) Income (low income defined in the context of local poverty lines)

b) Place of residence (rural and slum dwelling being associated with poverty)

c) Agency within a household (low agency being typically associated with female, gender and youth or old age)

(3) Deciding on appropriate study design(s): an inclusive approach toward individual study design was taken, but with explicit assessments of study quality against predefined criteria

(4) Identifying outcomes of interest: these were selected based on the global PPM objectives

(5) Process evaluation and understanding context: It was not possible to use process evaluation in this assessment, but context has been clearly acknowledged throughout

(6) Analysing and presenting data: the authors were unable to present aggregate data or meta-analyses, but have used the available data to make overall assessments

(7) Judging applicability of results: the results are applicable in relation to global PPM objectives.

## Findings

The findings are presented according to the different objectives of the review outlined above.

### Defining PPM for TB Care and Control - results from STOP-TB PPM subgroup literature and e-mail clarifications with the secretariat

As noted above, the original concept of PPM has evolved. The Stop TB concept: "*represent[s] a comprehensive approach to involve all relevant health-care providers in DOTS and ensure that they apply international standards for TB care, while taking on DOTS tasks according to their capacity. PPM DOTS targets a wide range of public as well as private health-care providers not yet sufficiently linked to NTPs. Depending on setting, these may include medical colleges, general hospitals, health services under specific insurance schemes, prison health systems, army health services, NGO health facilities, corporate health facilities, private specialists and general practitioners, private pharmacies and the informal private health-care sector"*. (DOTS Expansion Working Group Strategic Plan 2006-2015)[18].

The principles behind PPM for TB are: *"that the financial resources to establish and sustain the collaboration are provided or facilitated by the NTP, that drugs are provided free of charge or heavily subsidized, and that fees for tests and consultations are waived or kept to a minimum"*. (The Stop TB strategy WHO/HTM/TB/2006.368)[16]. Clarification about the PPM objectives was sought with the STOP-TB PPM subgroup secretariat in November 2009. This confirmed the four global objectives which were subsequently published in the PPM toolkit [17].

### Defining PPM for TB Care and Control - results from NTP manager interviews

At country level, the understanding and implementation of PPM activities varied considerably. For example in Pakistan only qualified private providers were involved in PPM activities; in Thailand the NTP worked together with a broad spectrum of care providers, including public hospitals, other public health care programs, the armed services, other workplaces and health insurers; and in Brazil TB care was only provided by the public sector but the private sector was used to ensure continued political support for the public TB services, to support social movement activities and to promote TB workplace activities.

Understanding of PPM by some NTP managers had also changed over time, as PPM itself evolved. Some NTP managers stated that their initial understanding of PPM was that it related to the private for-profit sector, although they had expanded to work with a number of different providers. One respondent stated "five years ago I was surprised when I came here [WHO Geneva] and was told that PPM also included other providers like public-public. I was of the opinion that PPM meant working with private for profit GPs only".

In many countries activities that could be considered PPM, in that they engage non-NTP actors, were not seen as such. For example in Cambodia, Myanmar and Mozambique the NTPs were engaging in partnerships with various types of community organizations and community providers, but did not recognize this as PPM and so did not report it as such.

### Mapping country-level partners and interventions

Most PPM publications referred to projects in Asia (Figure 2). Findings from the NTP manager interviews (Table 1) and from the publications (Table 2) were consistent in reporting that private for profit (PFP)-qualified clinical services (QCS) were the most frequent partner for their PPM programmes. The interviews (table 1) ranked the other public service (OPS) ministries providing communal living facilities (such as prisons and army barracks) second, while the NGO-QCS and the public sector health partners (PSHP)-QCS equally were the third most common partners. The publications (Table 2) ranked the NGO-QCS as the second and the NGO-unqualified providers as the third most common partner. Overall the NGO-QCS was the second most common type of partner.

It was clear that concepts of partnership varied. In some cases partners provided a limited range of services, while in others, they provided the majority. In Bangladesh, according to the NTP manager, BRAC provided TB support services to the general population in partnership with the NTP and additionally supervised 28 smaller NGO's and through them supported TB services in the army, police and prison system with resources and technical assistance.

The NTP managers (table 1) cited diagnosis, drug treatment and reporting as being equally common services while Table 2 shows that according to the literature, drug treatment was the service most frequently provided, followed jointly by diagnosis and reporting. Table 1 show that partners were supported by NTP inputs in the form of providing drugs and capacity building. The literature (table 2), also showed that drugs and capacity building were the most common NTP inputs.

### Reviewing practice against global PPM objectives

This was challenging due to the lack of systematic reporting of projects against the PPM objectives. Table 3 shows the characteristics of the study designs and the data presented in the project documents relating to the identified PFP- and NGO-QCS interventions. The number of projects reporting on case detection and treatment was higher than projects reporting on access, equity and cost reduction. Furthermore the quality of reported data, measured against our checklist, was also better for case detection and treatment outcome.

#### • Case detection

The definition of case detection was noted in the introduction. However there are a number of activities that contribute to case detection, not all of which require biomedical training. For example "active suspect identification" and "referral" are linked to the clinical aspect of case detection, but may be performed by individuals with a non-medical background [47]. Case detection was incorporated into PPM projects, but reported in sub-components such as advocacy, diagnosis or referral.

Four of the projects working with PFP-QCS had a control arm, and presented evidence that showed increased case detection. In New Delhi, India there was a near doubling of cases in the PPM project area compared to the control area[48,49]. In Myanmar the increase in both PPM project areas was higher than the contemporaneous increase in the control area. For example new smear positives rose from 46 to 85 per 100,000 between 2001 and 2004, compared to 23 to 36 per 100,000 over the same period in the control area[30,50,51]. In the study from Hyderabad, it is interesting to note that similar proportions of patients were referred from PFP- and NGO-QCS (42% and 46% respectively) while fewer were referred from PFP- Informal providers (33%). One study noted that case detection benefits can be achieved through the impact that engaging private providers can have on awareness of services: Newell et al.[52] found that after some time, many patients by-passed PFP-QCS and went directly to the DOTS centres linked to them, which were primarily NGO-run.

None of the NGO studies had a control arm, but there did appear to be an increase in case detection in before and after comparisons. Many of the NGOs were partners alongside PFP-QCS; it was not often clear what each of these contributed. In Indonesia[53] for example, the NGO facility diagnosed all the cases referred from multiple sources. Other studies noted increased case detection in the project areas, however it is not possible to directly attribute this to the intervention due to non-disaggregated data between provider types, and a lack of either a comparator area or a comparison with underlying trends in case detection.

It appears from some studies that increased case detection from PFP-QCS partners relied on the payment of incentives or on a mediating NGO and that when these were removed case detection declined[27-30,49,54-56].

#### • Treatment outcomes

Treatment success rates in most of the countries reporting PPM activities with PFP-QCS were generally high in both the public sector and with PPM partners, particularly NGOs (often over 85% and sometimes over 90%), providing little scope for substantial improvement. It is only in the projects with a control arm that any change can be attributed with reasonable certainty to the PPM interventions. In some countries treatment outcomes among private providers were poor and demonstrated marked improvement after inclusion in a PPM programme. For example, the New Delhi project[34] reported that the number of successfully treated cases (all categories) was higher in the PPM project (n = 204) than the non-DOTS treatment in the private sector (n = 121) by a factor of 69%. The treatment success rate (new smear positive) in the project was 81%, not statistically different from 86% in the Government Chest Clinic nor 82% in the broader public sector for New Delhi [34,48]. In Vietnam, treatment success rates among private providers improved from 49% to 62% through engagement in the PPM intervention, but remained substantially lower than the 87% achieved through the NTP[29,57]. In Nigeria[58] treatment success rates with the NGO partners (83.7%) and the public sector (78.6%) where not statistically significant different, but the default rates were lower with the NGO partner (5,8%) than in the public sector (13%).

The Myanmar Sun Quality Health (SQH) franchise[51] study provided an equity analysis on treatment outcomes and found that people with low socio-economic status had significantly lower treatment success rates than people from higher socio-economic groups within the project. Treatment success rates among higher socio-economic groups were 94% compared with 84% in lower socio-economic groups (P = 0.021)[51].

#### • Enhance access and equity

Some PPM interventions showed greater potential than others for increasing equity in access to TB services by being located predominantly in poor areas or serving poor population groups. Projects in Hyderabad,[31,34,59] Mumbai,[60,61] Myanmar,[50,51] South Africa[62] and Nepal[63] provided examples of these, while in the Philippines[64] the NGO was located in metro Manila which is a relatively wealthy location and in Kenya[65] the partners were located outside the slums.

Some studies provide more detailed equity analysis. For example the Myanmar SQH franchise[51] study showed that 48% of patients belonged to the lowest socio-economic quintile. The Bangalore[32,33] study presented the socio-economic profile of patients, showing that 50% had a low standard of living. It also showed that almost 50% of each of the lowest, middle and highest income groups where referred for TB diagnosis by a private practitioner. Total expenditure during care seeking for the lowest income group was US$120 vs. US$170 for high and medium income groups, however this constituted respectively 53% and 41% of annual household income per capita. Unfortunately data are not disaggregated between qualified and unqualified private providers. Some of the NGO-CQS projects were working with particularly marginalised groups in order to increase their access to TB services. In Egypt[54] for example, the NGO partner worked with African refugees who have poor access to public TB services. During the duration of the study in Nepal,[52,63,66] patients bypassed the PFP-QCS and went straight to the involved NGO hospital, similarly in Hyderabad[31,34,59] 51% of patients with suspected TB presented directly to the involved NGO hospital.

Many of the projects did not describe their location or provide information on the socioeconomic status of their patients. Some provided details of urban or rural location; in many countries rural areas are poorer, and it may be surmised that the rural projects in Thatta, Pakistan[56] and Pune[29,30,49,55] may have served mostly poor people. Others provided information on how efforts were made to increase access for the poor. One project in Nepal[52,63,66] for example aimed to ensure that patients did not have to travel more than 15 minutes to reach the nearest DOTS centre.

Some projects seemed less successful in reaching poorer patients, although the patients accessing services were by no means wealthy. In New Delhi[34,48], for example, the patients were predominately literate, lived in semi-urban areas, were employed in private establishments and belonged to the middle-income group, with an average monthly income of reported to be US$40.

Some studies reported gender differences in access to care and the change the projects made to this. For example in project areas in Hyderabad[31,34,59] the proportion of new smear positive women was reported to be higher than in the rest of Hyderabad (46% vs. 37%), unfortunately it was not possible to differentiate among the partners types to determine which made most contribution to the change.

#### • Reduce financial burden for patients

Among the few studies that reported against this objective, it appears that the costs to patients consulting PFP-QCS within a PPM programme were substantially less than those for patients visiting similar practitioners not engaged in the programme, the main reason being substantially lower expenditure on drugs [34]. For example, the New Delhi[34] study reported costs per patient visiting PFP-QCS involved in the PPM project as US$50-60, which, while still high, was approximately one third of the cost faced by patients consulting with private providers not involved in the project. In the two Myanmar studies[30,50,51] it was reported that costs were expected to be lower (one provided high levels of subsidies to partners), but actual costs were not provided.

A comparative study was undertaken in Hyderabad,[27,28,30,31,34,59,67] where it was reported that patients visiting PFP-QCS were diagnosed faster than in the public sector (8 vs. 10 weeks), paid less prior to diagnosis (US$5 compared with US$20), paid less during treatment (US$1 compared with US$11) and lost less income due to illness (1.4 against 2.8 months of lost wages). The total patient cost when attending a private partner of the PPM programme was US$50-60, while for non-DOTS treatment in the private sector, mean costs were US$111.

It appeared that the NGO projects tended to charge lower fees to patients. In the Egyptian[54] project, for example, drugs were provided for free and any patient needing inpatient care received it free of charge. Where there were charges sometimes part was refunded. In Timika[53] for example, patients were asked to deposit approximately US$20 which was paid back when the treatment was completed; there were exemptions for local patients of a specific ethnicity and for those who were very financially constrained.

Some studies had less positive findings. In some countries patients still pay for all or some aspects of diagnosis, even when private providers have been trained in DOTS (e.g. Kenya, [28-30,65] Chennai,[27,28,68] Kerala[30,31,35,44,69]). In some places, treatment is still charged for under PPM and is costly for patients and particularly for the poor. In Vietnam[57,70,71] it was stated that patients visiting PFP-QCS paid for treatment, but defaulted because they could not afford it: the monthly drug cost varied between US$12 and US$33 while 41% of patients earned less than US$40 per month. In many countries PFP-QCS continued to charge consultation fees.

Most studies however did not report patient costs, even fewer compared them to average patient incomes or socioeconomic status, and few of those that did report costs had a comparison arm. It is therefore not possible to say, for many projects, whether total costs for patients visiting the private sector have reduced as a result of PPM and whether these are lower than for patients visiting the public sector. Without an analysis of socioeconomic status it is not possible to say what proportion of average income is spent by patients accessing services.

## Discussion

'Engaging all care providers' is a strategy that can increase access to TB care and has done so in a number of countries. The strategy has the potential to do this for the poor and again, has done so in some places. It became clear through the interviews and the literature however that the breadth of PPM is not being explored in practice and that most projects currently are focussed on formal private for profit practitioners, with NGO qualified providers a secondary focus; with some evidence that NGO providers can offer greater gains in equity and patient cost reduction. Involvement of for-profit qualified providers are also most frequently mentioned in previous evaluations and cross project studies [27-31], however a systematic analysis of the implications of this has not been published.

The predominance of partnerships with qualified clinical service providers and particularly with private for profit practitioners is understandable given that the original motivations for PPM were to improve the quality of services received by patients who went to private providers. It is recognised, however that there is substantial country level interaction between public sector bodies under PPM, however it is not possible to assess the impact of these due to a lack of publications, which may be indicative of a lack of evaluations of output of such PPM setups. It is encouraging to see that efforts have been made to engage NGOs and it appears that this has contributed to access by poorer populations. There is however an array of other providers, particularly for-profit informal providers, who are accessed by the poor and who are not explicitly incorporated into PPM programmes. There does not appear to have been any assessment of the contribution they make to case detection, treatment success and increased equity in access to services. In some cases it seems that this is because they are considered by programme managers to be part of a community DOTS rather than a PPM approach. It was not possible to review the community DOTS literature as part of this review, thus it is not clear whether the role and effectiveness of for-profit informal providers is documented elsewhere.

An interesting finding from the interviews was that while partnerships with other parts of the public sector were clearly important and frequently conducted, they were not mentioned in the publications. There may be a number of reasons for this. Some evaluations excluded this type of PPM intervention, either explicitly [31] or through defining PPM as the link between the national TB programme and private for-profit providers and NGOs [27-30]. Another reason is that NTP managers have formal links with other parts of the public sector through the Minister of Health who will hold discussions with the heads of other ministries at cabinet level. It may be that the limited need for technical assistance also means that there are fewer publications about public-public partnerships.

It could be argued that the limited range of partner types is unimportant since one of the principles of PPM is that the objectives are defined locally. However it does appear from the evidence that choice of partner affects the outcomes of PPM interventions in terms of case detection, treatment success, access by different socio-economic groups and poverty alleviation. In the projects that have been able to demonstrate increased access for poor and other marginalised people it is clear that the designers and implementers strategically chose partners to which poor people have geographical, social and (sometimes) financial access. In many sites this was done through reducing the economic cost to patients (in terms of money and also time spent care seeking); in Egypt a new service was created for those who could not access the public health services.

Most projects report some level of geographical information (e.g. rural, urban, peri-urban), sometimes as a proxy for poverty. Few provide data on distances travelled by patients to access services by different types of providers. In future studies Geographic Information Systems techniques may be used to support spatial mapping of providers, which could help with partner selection and provide information about the equity of interventions through identifying whether quality services are being taken closer to patients [72-74].

With the exception of using geographical markers, it was difficult to draw conclusions about whether the selected partners increased equity in access to services, due to the limited availability of socioeconomic data. In another assessment, Lönnroth et al. found that only one out of four studied projects could be said to increase access and equity [29].

### Limitations

The authors recognise a number of limitations to the review methodology as well as limitations to the availability and analysis of evidence.

The first limitation is the lack of standardised methods for assessing complex interventions. As noted above, several assessment frameworks were considered, but none were suited to providing a basis for assessing complex interventions using mixed methods research. Furthermore, the method we chose for identifying the predominant types of interventions is open to criticism. We used a scoring system to assess the frequency with which partners were engaged by NTPs as reported by NTP managers and in the published literature. While such a method provides an approximation of the types of PPM models in practice it does not capture the importance of the different elements of the project, for example whether it is weighted toward case detection or to improving quality of treatment. Further we were only able to analyse the detail of the projects among the predominant types of partner. It may be that some projects using less frequently reported partners had better outcomes in terms of case detection or equity.

As noted in the methods, assessing equity was challenging. Most project evaluations did not consider differences in socio-economic status of the patients visiting different types of provider. When programmes involved more than one type of for-profit provider the data were not spilt between these providers (for example qualified and unqualified practitioners), nor was this seen as a concern within those evaluations [27-31].

The review was also limited with regard to the way it was able to assess the contributions of multiple partners to a single project; for example, we have not been able to explore the contributions of different agents, such as medical associations. We focussed instead on the partners with whom patients have direct contact. This limitation arose largely from the lack of detail in publications, however we do not see this as a significant limitation, since it is the contact partner (e.g. the private-for profit practitioner) that most influences the patient's experience of care.

There were also limitations relating to data availability. Some (unpublished) reports were not available to the authors; we were also only able to interview 12/22 NTP managers from high burden countries. Both these concerns mean that some forms of partnership may not have been included in the analysis. However from the data available and the consistency of engagement particularly regarding partner type, we do not believe that the conclusions would have changed substantially.

## Conclusion

PPM has had success in improving case detection and treatment outcomes among patients seeking care with private partners. Evidence for reduced costs for the patients is inconclusive however, and there is still scope for increasing equity in access to care, by systematically engaging providers who are the primary agents for poor people seeking health care.

Guidelines outlining which types of providers best contribute to achieving the four global objectives, along with the resources (financial, human and other) required by NTPs for such engagement would assist with decisions about which type of providers to engage.

It would be useful if evaluators of programmes systematically consider all the four global PPM objectives in their analyses: if equity is being measured then the chances of it being delivered improve also. Such analysis would help policy makers at national and international level identify gaps in service provision and think creatively about how to address them.

## Competing interests

All authors have completed the Unified Competing interest form at http://www.icmje.org/coi_disclosure.pdf (available on request from the corresponding author) and declare that (1) RM, has support from LHL for the submitted work; (2) RM, GM, SBS have no relationships with companies that might have an interest in the submitted work in the previous 3 years; (3) their spouses, partners, or children have no financial relationships that may be relevant to the submitted work; and (4) RM, GH, SBS have no non-financial interests that may be relevant to the submitted work.

## Authors' contributions

All authors developed the idea for this article. RM produced the first draft of the article, all authors prepared the final draft and all authors read and approved the final manuscript. SBS is guarantor.

## Supplementary Material

Additional file 1 **Overview of providers and aggregated provider groups**. The table shows all the named providers in the interviews with the National Tuberculosis Programme managers, in addition to showing which of the individual providers make up each of the aggregated provider groups used in table 1 and 2. Click here for file

Additional file 2 **Overview of service provided by providers**. The table shows all the types of service provided by the providers in addition to showing which of the individual service make up each of the aggregated provider service used in table 1 and 2. Click here for file

Additional file 3 **Overview of National Tuberculosis Programme inputs received by provider**. The table shows all the types of National Tuberculosis Program inputs received by providers in addition to showing which of the individual inputs make up each of the aggregated National Tuberculosis Programme inputs used in table 1 and 2. Click here for file
