# Supplementary material for: A systematic assessment of the concept and practice of public-private mix for tuberculosis care and control
Source: Int J Equity Health. 2011 Nov 10;10:49. doi: 10.1186/1475-9276-10-49 (PMC3238294; doi:10.1186/1475-9276-10-49)
Supplement: Additional file 1 — Overview of providers and aggregated provider groups. The table shows all the named providers in the interviews with the National Tuberculosis Programme managers, in addition to showing which of the individual providers make up each of the aggregated provider groups used in table 1 and 2. [file 1475-9276-10-49-S1.DOC]

**Additional file 1: Providers and aggregated provider groups**

| **Broad partner categories** | **Providers noted in interviews** | **Aggregated provider groups** |
| --- | --- | --- |
| **Public Sector Health Partners (PSHP)** | Tertiary, district hospitals | Qualified Clinical Services |
|  | District hosp. General hosp. |  |
|  | Health offices, district, provincial, primary |  |
|  | Infectious disease hospitals | Specialist referral services |
|  | Lung clinics |  |
|  | University hosp./ Teaching hosp./medical college |  |
|  | Other PHC^a^ programmes. HIV/AIDS^b^, Malaria | Other vertical programmes |
|  | Family Health programmes |  |
|  | Reproductive health |  |
|  | Semi-public hospital - joint public sector, international NGOs | Semi-public services |
|  |  |  |
| **Other Public Sector (OPS)** | Other ministries; air force, army, navy, police, prison | Ministries providing communal living facilities |
|  | Ministry of Home Affairs |  |
|  | Ministry of justice |  |
|  | Ministry of social security | Ministry of social security |
|  | Health insurance | Health insurance |
|  | Ministry of local government and rural development & cooperation | Other ministries |
|  | Local government |  |
|  | Ministry of Vocational training |  |
|  | Ministry of Labour |  |
|  | Ministry of Information |  |
|  | Food and drug administration |  |
|  | Parastatals | Parastatals |
|  | Ministry of transport (Railroads) |  |
|  |  |  |
| **Private For-profit (PFP)** | G.P.^c^ with own clinic, Nurses with own clinics, hospitals, polyclinics | Qualified Clinical Services |
|  | Qualified pharmacists | Qualified ancillary health services |
|  | Laboratories |  |
|  | Grocers, traditional healers, non-qualified providers, drug vendors | Informal Providers |
|  | Semi-qualified practitioners with own clinic |  |
|  | Federation of employ. &employees | Workplace programmes |
|  | Confederation of industry and commerce |  |
|  | Garment Industry confederation |  |
|  | Private sector workplace |  |
|  |  |  |
| **Non Governmental Organisations (NGO)s** | Health service implementers, NGO clinics, hospitals | Qualified Clinical Services |
|  | Charity hospitals |  |
|  | Faith based clinics and hospitals |  |
|  | Trade Union hospitals |  |
|  | Health service centres + laboratories |  |
|  | Social marketing NGO's | International NGOs |
|  | International NGO's |  |
|  | For-profit NGO's Aga Khan Health services |  |
|  | Community CBO's^d^, patient groups | Informal unqualified providers* |
|  | Volunteer health workers |  |
|  | Local NGO's |  |
|  | Family member |  |

*Qualification for the purpose of this table implies a formal, recognised qualification in a branch of biomedical healthcare provision

a PHC = Public Health Care

b HIV/AIDS = Human immunodeficiency virus/ Acquired Immune Deficiency Syndrome

c G.P. = General Practitioners

d CBO’s = Community Based Organisations
