# Supplementary material for: A systematic assessment of the concept and practice of public-private mix for tuberculosis care and control
Source: Int J Equity Health. 2011 Nov 10;10:49. doi: 10.1186/1475-9276-10-49 (PMC3238294; doi:10.1186/1475-9276-10-49)
Supplement: Additional file 2 — Overview of service provided by providers. The table shows all the types of service provided by the providers in addition to showing which of the individual service make up each of the aggregated provider service used in table 1 and 2. [file 1475-9276-10-49-S2.DOC]

**Additional file 2: Service provided by providers**

Ordered according to patient pathway

| **Original provider service** | **Aggregated provider service** |
| --- | --- |
| Advocacy | Advocacy |
| Case detection | Active Suspect identification* |
| Diagnosis | Diagnosis |
| Referral | Referral† |
| Treatment | Treatment |
| Health education | Health education |
| Report | Report |
| Patient follow up/ DOT^a^ provider | DOT^a^ |
| Treatment support | Treatment support |
| Free transport |
| Food baskets |
| Default tracing | Default tracing |
| Political lobbying | Political lobbying |
| Supervision of Work places | Supervision of Work places |

* On discussion with the national tuberculosis program (NTP) managers it became clear that

‘case detection’ involved active suspect identification and referral.

† referral following patient care seeking

a DOT = directly observed treatment
