# Supplementary material for: A systematic assessment of the concept and practice of public-private mix for tuberculosis care and control
Source: Int J Equity Health. 2011 Nov 10;10:49. doi: 10.1186/1475-9276-10-49 (PMC3238294; doi:10.1186/1475-9276-10-49)
Supplement: Additional file 3 — Overview of National Tuberculosis Programme inputs received by provider. The table shows all the types of National Tuberculosis Program inputs received by providers in addition to showing which of the individual inputs make up each of the aggregated National Tuberculosis Programme inputs used in table 1 and 2. [file 1475-9276-10-49-S3.DOC]

**Additional file 3: National tuberculosis program inputs received by provider**

| **Original NTP^a^ inputs** | **Aggregated NTP^a^ inputs** |
| --- | --- |
| ACSM^b^ activities | ACSM^b^ activities/ IEC^c^ materials |
| IEC^c^ material |
| BCG^d^ vaccine | BCG^d^ vaccine |
| Diagnosis | Diagnosis |
| Drugs | Drugs |
| Drug boxes |
| Default tracing | Default tracing |
| Pay for service (reimbursement scheme) | Pay for service (reimbursement scheme) |
| Lab equipment | Diagnostic supplies |
| X-ray film |
| Sputum cups |
| Reporting forms | Monitoring supplies |
| Requisition forms |
| Treatment cards |
| Registers |
| Supervision | Supervision |
| Training | Capacity building |
| Capacity building |
| Transport | Transport |
| TB-clinic (building) | TB-clinic (building) |

a NTP = National Tuberculosis Program

b ACSM = Advocacy, Communication and Social Mobilisation

c IEC = Information, Education and Communication

d BCG = Bacille Calmette Guérin vaccine
